# The Kidney–Brain Axis in Chronic Kidney Disease: Uremic Toxins, Cognitive Decline, Mechanistic Pathways, Biomarkers and Therapeutic Perspectives

**DOI:** 10.3390/biomedicines14071579

**Published:** 2026-07-15

**Authors:** Valentino Rački, Božidar Vujičić, Vita Komen, Lara Saftić Martinović, Nada Birkić, Ivan Bubić, Almir Fajkić, Andrej Belančić

**Affiliations:** 1 Department of Neurology, Faculty of Medicine, University of Rijeka, Braće Branchetta 20, 51000 Rijeka, Croatia; 2 Department of Internal Medicine, Faculty of Medicine, University of Rijeka, Braće Branchetta 20, 51000 Rijeka, Croatia; bozidar.vujicic@uniri.hr (B.V.);; 3 Department of Medical Biology and Genetics, Faculty of Medicine, University of Rijeka, Braće Branchetta 20, 51000 Rijeka, Croatia; 4 Faculty of Biotechnology and Drug Development, University of Rijeka, Radmile Matejčić 2, 51000 Rijeka, Croatia; 5 Department of Pathophysiology, Faculty of Medicine, University of Sarajevo, 71000 Sarajevo, Bosnia and Herzegovina; 6 Department of Basic and Clinical Pharmacology with Toxicology, Faculty of Medicine, University of Rijeka, Braće Branchetta 20, 51000 Rijeka, Croatia

**Keywords:** chronic kidney disease, cognitive impairment, uremic toxins, kidney–brain axis, neuroinflammation

## Abstract

Chronic kidney disease is increasingly recognised as a systemic disorder with important neurological consequences, including cognitive impairment. However, the field remains challenging because CKD-related cognitive decline involves diverse uremic toxins, overlapping vascular, inflammatory, metabolic, endothelial, and neurodegenerative pathways, inconsistent cognitive screening practices, and no unified treatment strategy. Within this context, the kidney–brain axis provides a useful framework for integrating renal dysfunction, toxin retention, systemic inflammation, blood–brain barrier disruption, and cognitive vulnerability. Of these mechanisms, uremic neurotoxicity offers a biologically credible connection between compromised renal clearance and cerebral dysfunction. Retained solutes such as indoxyl sulfate, p-cresyl sulfate, indole-3-acetic acid, trimethylamine-N-oxide, urea, guanidino compounds, lanthionine, quinolinic acid, and homocysteine may induce endothelial injury, oxidative stress, neuroinflammation, mitochondrial dysfunction, excitotoxicity, and glial activation. Although these pathways are supported by experimental and translational studies, direct causal evidence in humans remains limited, and most clinical data should currently be interpreted as associative rather than definitive proof of causality. These processes converge on neuronal and synaptic vulnerability and may elucidate the distinctive cognitive profile associated with chronic kidney disease, particularly deficits in attention, processing speed, and executive function. This review summarizes the most recent evidence on the epidemiology and clinical phenotype of cognitive impairment in chronic kidney disease. It also discusses the molecular and cellular mechanisms of uremic neurotoxicity and examines new biomarkers of the kidney–brain axis, including neurofilament light chain, glial fibrillary acidic protein, brain-derived neurotrophic factor, tight junction proteins, and uremic toxins. However, these biomarkers remain insufficiently validated for routine clinical use, as their interpretation is complicated by reduced renal clearance, systemic inflammation, comorbid vascular disease, methodological heterogeneity, and the lack of longitudinal studies linking biomarker changes to cognitive outcomes. Therapeutic strategies targeting uremic toxins remain compelling from a mechanistic standpoint, but they are not yet fully developed in clinical practice. Subsequent research ought to amalgamate toxin profiling, cognitive phenotyping, neuroimaging, endothelial and inflammatory biomarkers, alongside patient-centered outcomes. Integrating cognitive assessment into nephrology care may enhance risk stratification, collaborative decision-making, and personalised management for patients with chronic kidney disease.

## 1. Introduction

Cognitive impairment (CI) is being more recognised as one of the important neurological complications of chronic kidney disease (CKD). Traditionally, CKD has been viewed mainly through its renal, cardiovascular and metabolic consequences. However, declining kidney function also appears to affect brain health, cognitive performance, and functional independence. Cognitive impairment is defined as a measurable decline in one or more cognitive domains, including memory, attention, processing speed, executive function, language, and visuospatial abilities [1]. Mild cognitive impairment is an intermediate state in which cognitive performance is reduced but daily independence is preserved, while dementia implies more severe and progressive impairment with loss of functional autonomy. In CKD, the cognitive profile often includes deficits in attention, processing speed, memory, and executive function, all of which are important for treatment adherence, medication management, dietary restrictions, dialysis planning, and shared decision-making [2].

### 1.1. Brain Health and CKD

The importance of cognitive decline in CKD should also be understood within the wider concept of brain health. Brain health is not limited to the absence of dementia or neurological disease [3]. It reflects the brain’s capacity to maintain cognitive, emotional, behavioural, sensory, and motor functions throughout a person’s life. This perspective shifts attention from late recognition of established cognitive disorders toward prevention, early detection and preservation of cognitive reserve [4]. Brain health becomes especially vulnerable in older individuals, as biological ageing, vascular injury, chronic inflammation, psychological stress, and various comorbidities converge. CKD represents a clinical condition in which many of these processes are present simultaneously and can be considered a marker of reduced brain resilience and a determinant of future brain health outcomes [2].

### 1.2. Epidemiology

The epidemiological burden of CKD-related cognitive impairment is substantial. More than 800 million people worldwide live with CKD, and several million require chronic renal replacement therapy for end-stage kidney disease [5]. Cognitive impairment is more common in CKD patients than in the general population, with the highest prevalence observed in patients receiving dialysis. Current estimates put cognitive impairment at approximately 10–40% of CKD patients and depend on age, disease stage and assessment method. Pooled data indicate an overall prevalence of about 40%, with 50% among hemodialysis patients, and it remains clinically relevant in peritoneal dialysis, non-dialysis end-stage kidney disease, and kidney transplant populations [6]. Earlier reviews similarly reported cognitive impairment or overt dementia in 20–50% of older patients with moderate CKD and in up to 70% of patients with severe CKD or dialysis dependence [2].

The wide variation in reported prevalence should be interpreted considering substantial methodological and clinical heterogeneity across studies. Estimates differ according to publication period, study design, geographical region, age distribution, educational background, comorbidity burden, CKD stage, dialysis status, dialysis vintage, and dialysis modality. In addition, cognitive impairment has been assessed using different screening instruments and diagnostic thresholds, including global tools such as the Mini-Mental State Examination (MMSE) and Montreal Cognitive Assessment (MoCA), as well as more detailed neuropsychological batteries. These differences are particularly relevant in CKD, where executive dysfunction, attention deficits, and reduced processing speed may be more prominent than isolated memory impairment. Therefore, the reported prevalence estimates should not be interpreted as directly interchangeable, but rather as reflecting heterogeneous populations, assessment strategies, and clinical settings.

### 1.3. CKD and Cognitive Impairment

The relationship between CKD and cognitive decline appears to follow a severity gradient, although the contribution of individual kidney markers is not uniform across studies. Lower estimated glomerular filtration rate (eGFR), albuminuria, proteinuria, higher serum creatinine and acute kidney injury have all been associated with increased risk of cognitive decline or dementia. A systematic review and meta-analysis of longitudinal studies found that CKD, acute kidney injury, higher serum creatinine, higher urinary albumin-to-creatinine ratio and lower eGFR were associated with a higher risk of dementia or cognitive decline, although the authors also emphasised heterogeneity and the need for stronger evidence [7]. More recent prospective data from the Chronic Renal Insufficiency Cohort showed that a higher urinary protein-to-creatinine ratio was associated with impairment in attention, processing speed and executive function, while the combination of reduced eGFR and increased proteinuria was associated with a higher risk of global cognitive impairment [8]. These findings support the view that CKD severity is clinically relevant to brain outcomes and suggest that proteinuria and vascular endothelial injury may be especially important markers of risk.

Cognitive impairment in all diseases affects quality of life [9]. Cognitive impairment can make it harder to do everyday tasks, make people less independent, make it harder to interact with others, and make people more likely to become depressed or anxious. It might also make it harder to follow the rules for managing CKD, such as taking medications on time, attending dialysis sessions, following dietary restrictions, and contacting doctors promptly. In populations undergoing dialysis, cognitive impairment is associated with elevated hospitalisation rates, increased healthcare utilisation, and suboptimal overall outcomes. Cognitive impairment in CKD is frequently overlooked and inadequately addressed, primarily due to the absence of consistent cognitive screening and structured assessment and intervention pathways in routine nephrology care [6].

The kidney–brain axis provides a valuable biological framework for understanding the potential acceleration of cognitive decline in CKD. In CKD, the kidney-to-brain component of this axis is driven by several interrelated pathological processes. Reduced renal clearance leads to the retention of uremic toxins, including protein-bound and gut-derived solutes, which may promote endothelial dysfunction, oxidative stress, systemic inflammation, blood–brain barrier disruption, and neuroinflammatory activation. These processes are further amplified by CKD-associated vascular calcification, arterial stiffness, anemia, hypertension, and impaired cerebral perfusion, thereby increasing vulnerability of frontal–subcortical networks involved in attention, processing speed, and executive function. Conversely, the brain-to-kidney component may involve autonomic dysregulation, sympathetic overactivity, neuroendocrine stress responses, altered renal perfusion, and impaired sodium and fluid homeostasis. Within this bidirectional framework, uremic toxins represent a core biological link because they connect impaired renal excretion with vascular, inflammatory, metabolic, and barrier-related mechanisms that may affect brain function [10]. This idea is helpful in the clinic because it integrates vascular, metabolic, and neuroinflammatory mechanisms into a single model of cognitive vulnerability related to CKD.

## 2. Methods: Literature Search and Conceptual Framework

### 2.1. Narrative Review Methodology

This article was designed as a narrative review with scoping elements to integrate epidemiological, mechanistic, translational, and clinical evidence regarding cognitive impairment in chronic kidney disease (CKD), with a particular focus on the role of uremic toxins and the kidney–brain axis.

Given the multidisciplinary nature of the field, encompassing nephrology, neurology, vascular biology, neuroimmunology, microbiome research, and biomarker science, the objective was to synthesise concepts across diverse areas of investigation rather than undertake quantitative pooling or formal systematic evaluation. This approach was considered most appropriate for developing a comprehensive, hypothesis-driven overview of the mechanisms linking impaired renal function with cognitive decline and for identifying emerging therapeutic opportunities targeting uremic neurotoxicity.

### 2.2. Information Sources and Search Period

A targeted literature search was conducted using PubMed and Scopus. To maximise coverage and identify additional relevant publications, manual screening of reference lists from key articles, reviews, consensus statements, and original studies was also performed.

The search included publications from January 2011 to 15 April 2026, corresponding to the period during which substantial advances were made in the understanding of the kidney–brain axis, uremic neurotoxicity, neuroimaging, biomarker development, and therapeutic strategies targeting uremic toxins.

Seminal historical publications published before 2011 were included selectively when considered essential for contextualising foundational concepts, including the classification of uremic toxins, mechanisms of blood–brain barrier dysfunction, neuroinflammatory pathways, and the evolution of cognitive assessment in CKD.

### 2.3. Search Strategy and Eligibility Criteria

Search terms combined controlled vocabulary and free-text keywords related to CKD, cognitive impairment, uremic toxins, biomarkers, and the kidney–brain axis. Representative search terms included: “chronic kidney disease”, “end-stage kidney disease”, “dialysis”, “cognitive impairment”, “cognitive decline”, “dementia”, “kidney–brain axis”, “uremic toxins”, “indoxyl sulfate”, “p-cresyl sulfate”, “indole-3-acetic acid”, “trimethylamine N-oxide”, “homocysteine”, “quinolinic acid”, “blood–brain barrier”, “neuroinflammation”, “oxidative stress”, “glymphatic system”, “neurofilament light chain”, “glial fibrillary acidic protein”, and “brain-derived neurotrophic factor”.

Articles were considered eligible if they fulfilled one or more of the following criteria:

(i) Reported epidemiological data or characterised cognitive phenotypes associated with CKD;
(ii) Investigated molecular, cellular, or experimental mechanisms linking renal dysfunction to cognitive impairment;
(iii) Evaluated biomarkers relevant to the kidney–brain axis, including markers of neuroaxonal injury, glial activation, endothelial dysfunction, blood–brain barrier integrity, or uremic toxin burden;
(iv) Examined therapeutic strategies targeting uremic toxins or interventions relevant to cognitive outcomes in CKD;
(v) Provided conceptual, translational, or historical insights necessary to contextualise the relationship between CKD, uremic neurotoxicity, and cognitive decline.

Eligible study designs included preclinical investigations, in vitro studies, animal models, observational studies, cohort studies, clinical trials, translational studies, metabolomic analyses, systematic reviews, meta-analyses, and relevant consensus documents. Publications not available in English and studies unrelated to CKD, cognitive impairment, the kidney–brain axis, or uremic toxin biology were excluded. The relevance and inclusion of references were determined by team consensus, involving a nephrologist (B.V. and I.B.), neurologist (V.R.), clinical pharmacologist (A.B.), and an expert in preclinical models and pathophysiology (A.F.), with L.S.M. acting as arbiter in cases of disagreement.

### 2.4. Evidence Synthesis and Limitations

Evidence from epidemiological, mechanistic, biomarker, and clinical studies was synthesised thematically to integrate biological mechanisms with clinical manifestations and potential therapeutic implications. The narrative synthesis was structured around five interconnected domains: (i) epidemiology and cognitive phenotypes in CKD; (ii) molecular and cellular mechanisms of uremic neurotoxicity; (iii) biomarkers of the kidney–brain axis; (iv) therapeutic strategies targeting uremic toxins; and (v) future directions for translational research and clinical implementation.

This approach facilitated the identification of recurring patterns, areas of convergence between experimental and clinical evidence, and key knowledge gaps relevant to precision nephrology and cognitive health. As a narrative review, this work did not adhere to formal PRISMA methodology and no structured risk-of-bias assessment was performed. Although predefined search procedures and eligibility criteria were used to enhance transparency and reproducibility, the heterogeneity of study designs, patient populations, biomarkers, and cognitive outcomes limited the feasibility of quantitative synthesis. Nevertheless, a narrative approach was considered appropriate for integrating mechanistic and clinical evidence across a rapidly evolving and inherently interdisciplinary field, where the principal objective was to generate a coherent conceptual framework linking uremic toxins, the kidney–brain axis, and cognitive decline in CKD.

## 3. Molecular and Cellular Mechanisms of Uremic Neurotoxicity

The mechanisms linking CKD to cognitive decline are multifactorial, but uremic neurotoxicity provides one of the most plausible explanations for direct kidney–brain interaction [11]. Impaired glomerular filtration and tubular secretion lead to the retention of a range of solutes that would normally be eliminated in urine. These compounds differ in molecular size, protein binding, lipid solubility, and origin, and include small water-soluble molecules, medium-sized molecules, and protein-bound toxins derived from host metabolism, diet, and the gut microbiome [12]. Their effects are not limited to the kidney or vascular system; accumulating evidence suggests that they can influence endothelial function, blood–brain barrier integrity, glial activation, oxidative stress and neuronal signalling. Therefore, uremic neurotoxicity should be understood as the cumulative effect of a disturbed internal biochemical milieu on the brain and its barrier [13].

However, it is important to distinguish mechanistic plausibility from established causality. Much of the evidence relating uremic toxins, oxidative stress, neuroinflammation, endothelial dysfunction, and blood–brain barrier disruption to cognitive impairment is based on in vitro, animal, and translational experimental studies. Human studies generally support associations between CKD severity, circulating biomarkers, vascular injury, inflammatory markers, uremic toxin levels and cognitive performance, but do not always establish temporal sequence or direct causation. Hence, the mechanisms discussed below should be seen as biologically plausible and potentially contributing pathways, rather than fully established causal mechanisms of cognitive decline in humans with CKD.

**Figure 1:**
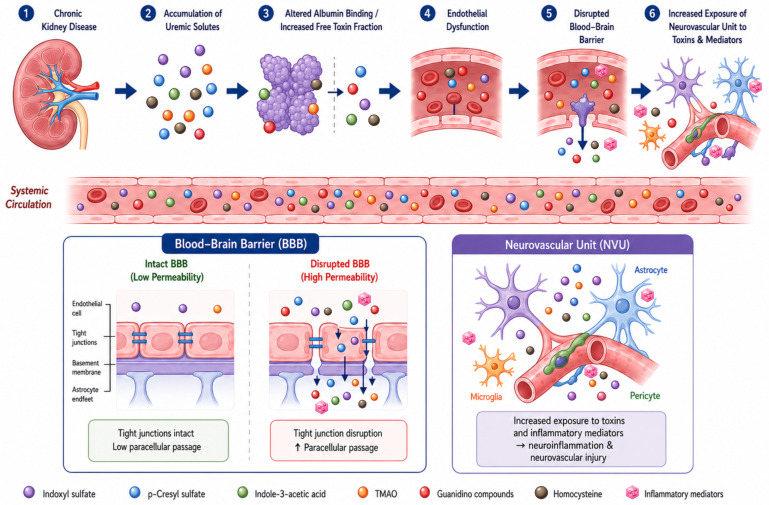
Renal toxin retention and blood–brain barrier disruption in chronic kidney disease. Reduced renal filtration and tubular secretion promote the accumulation of circulating uremic solutes. Altered albumin binding, endothelial dysfunction, and tight-junction disruption increase blood–brain barrier permeability, facilitating greater exposure of the neurovascular unit to uremic toxins and inflammatory mediators (Created in BioRender. FAJKIĆ, A. (2026) https://BioRender.com/uvbgpkh).

A major question in the field is how circulating uremic toxins reach the central nervous system or affect it. Under physiological conditions, the blood–brain barrier (BBB) limits the entry of many circulating substances, especially protein-bound toxins bound to albumin [12]. However, CKD alters this protection in several ways. Firstly, uremia reduces albumin’s ability to bind toxins, increasing the free fraction of toxins and potentially enhancing their biological activity. Secondly, CKD is associated with endothelial dysfunction and structural alterations in tight junction proteins, which may increase BBB permeability. Experimental and limited clinical observations suggest that toxins such as indoxyl sulfate and trimethylamine-N-oxide (TMAO) can compromise barrier integrity, thereby increasing the brain’s exposure to circulating inflammatory mediators and neurotoxic solutes [12,14]. The sequence from renal toxin retention to blood–brain barrier disruption is summarised in Figure 1.

### 3.1. Uremic Toxins

Among individual toxins, indoxyl sulfate has received particular attention. It is a protein-bound toxin derived from gut microbial metabolism of tryptophan and is markedly retained in CKD [12]. Although many uremic toxins converge on shared downstream pathways such as oxidative stress, endothelial dysfunction, inflammation, and impaired neuronal signaling, their primary pathogenic positions are not identical [12,13]. Indoxyl sulfate appears most closely linked to endothelial injury, AhR-mediated signaling, oxidative stress, and blood–brain barrier dysfunction [15,16,17]; p-cresyl sulfate is mainly associated with oxidative stress, mitochondrial dysfunction, and vascular inflammation; guanidino compounds primarily affect excitatory–inhibitory neurotransmitter balance through N-methyl-D-aspartate (NMDA) receptor activation and impaired GABAergic signaling [18,19,20]; homocysteine contributes mainly through endothelial dysfunction, prothrombotic effects, oxidative stress, and excitotoxicity [21,22,23,24]; and quinolinic acid links altered tryptophan metabolism with NMDA receptor-mediated excitotoxicity and neuroinflammation [25,26,27,28].

Experimental studies suggest that indoxyl sulfate can cross a disrupted BBB and exert direct effects on neural and vascular cells [29]. In neuronal models, it reduces cell viability and glutathione availability, while increasing oxidative stress, inflammation and cell death [12]. In animal models of CKD, indoxyl sulfate has been detected in brain tissue and has been associated with neurobehavioral abnormalities [29], although it is important to highlight that clinical observations remain limited. These observations are significant as they link gut dysbiosis, compromised renal clearance, and brain dysfunction within a singular mechanistic framework.

Other retained solutes may contribute through distinct but overlapping mechanisms. Patients with CKD can have too many guanidino compounds in their brains, which can mess with neurotransmission. These compounds include guanidine, guanidinosuccinic acid, methylguanidine, and creatinine. Their neurotoxic effects are partly related to altered glutamatergic and GABAergic signalling. By activating NMDA receptors and weakening inhibitory GABAergic tone, these compounds may promote calcium influx, excitotoxicity, nitric oxide production and excessive glutamate release [30,31]. Homocysteine, another retained protein-bound toxin, may contribute to cognitive dysfunction through disruption of methionine metabolism, oxidative stress, epigenetic dysregulation, protein dysfunction and endothelial injury [21,22]. In uremia, modified protein binding may elevate the biologically active free fraction of homocysteine, whereas interactions between guanidino compounds and homocysteine binding to albumin may enhance neurotoxic exposure [32,33]. Uremic toxins are covered in more detail further in the manuscript.

### 3.2. Tryptophan Metabolism

Another important pathway is tryptophan metabolism. Kynurenic acid and quinolinic acid are metabolites of the kynurenine pathway that have opposite effects on neuronal function or effects that depend on the situation. Kynurenic acid functions as a neuronal receptor antagonist and may exhibit neuroprotective effects by modulating glutamate binding and neuroexcitatory signalling; however, its concentration in the central nervous system is limited by the blood–brain barrier [34,35]. Kynurenic acid analogues have been investigated in experimental models of neurodegenerative diseases, such as Alzheimer’s disease and Huntington’s disease, underscoring the broader significance of this pathway in neuronal resilience [36,37]. Quinolinic acid, on the other hand, works as an NMDA receptor agonist and can be dangerous if there is too much of it or if it is not cleared properly. It does not readily cross an intact blood–brain barrier, but it can enter the central nervous system when the barrier is damaged, leading to long-term NMDA receptor activation, excitotoxicity, and neuronal damage [25,26,27]. This pathway is important because learning and memory depend on glutamatergic signalling that is carefully controlled. Too little or too much NMDA receptor activity can make synaptic plasticity worse [12].

### 3.3. Oxidative Stress and Mitochondrial Dysfunction

Oxidative stress is a biologically plausible cellular process through which uremic toxins may affect the brain. CKD has been associated with higher levels of reactive oxygen species and weaker antioxidant defences. This imbalance in redox reactions damages blood vessels, reduces endothelial function, and accelerates cellular ageing [38,39]. Oxidative stress in the brain can harm lipids, proteins, and DNA, disrupt mitochondrial function, and make neurons less resilient, thereby accelerating neurodegenerative processes and cognitive decline [40]. Indoxyl sulfate and associated toxins can diminish glutathione availability and stimulate inflammatory transcriptional pathways, such as nuclear factor-κB signalling in glial cells, while also exacerbating oxidative stress and endothelial dysfunction [41,42]. Mitochondrial dysfunction is particularly significant given the high energy demands of neurons and their limited capacity to withstand metabolic stress. Experimental CKD models support a link between kidney failure, mitochondrial dysfunction, oxidative stress, inflammation and cognitive abnormalities [43,44]. Mitochondrial dysfunction can exacerbate uremic neurotoxicity via several interlinked mechanisms. Neurons largely rely on oxidative phosphorylation for ATP production. Consequently, impaired mitochondrial respiration can result in neuronal energy failure, reduced synaptic efficacy, and impaired maintenance of ion gradients [45]. Energy depletion may compromise Na+/K+-ATPase activity, disrupt calcium homeostasis and enhance susceptibility to excitotoxic injury, particularly in conjunction with changes in glutamatergic signaling and reduced inhibitory tone [43]. At the same time, defective mitochondria are another source of reactive oxygen species, thereby creating a vicious cycle where oxidative stress damages mitochondrial proteins, mitochondrial DNA and membrane potential further [46]. In CKD, retained uremic toxins may also affect mitochondrial function, antioxidant defenses, endothelial metabolism, and glial activation, thereby linking metabolic stress with blood–brain barrier dysfunction, neuroinflammation, and synaptic vulnerability [47,48]. Thus, mitochondrial dysfunction should not be viewed only as a downstream consequence of oxidative stress, but also as an active amplifier of neuronal energy failure and toxin-mediated brain injury. When oxidative damage and mitochondrial dysfunction affects neurons, endothelial cells, and glial cells simultaneously, it sets off a cycle of vascular dysfunction, neuroinflammation, and impaired synaptic activity that progressively worsens [12].

### 3.4. Neuroinflammation

Oxidative stress and neuroinflammation are closely linked. Chronic low-grade systemic inflammation, immune cell dysfunction, and increased production of inflammatory mediators are all signs of CKD [10,49]. Uremia is characterised by modifications in immune cell composition and functionality, including a diminished lymphoid cell count and activity, an expansion or activation of myeloid populations, and an elevated production of inflammatory cytokines and reactive oxygen species [12]. Experimental data suggest that when the blood–brain barrier is not working properly, circulating cytokines, immune cells, and uremic toxins may have a more direct effect on the brain [50]. Microglia, the resident immune cells of the central nervous system, regulate synaptic function and cognitive processes, but persistent activation can lead to the release of cytokines, chemokines, reactive oxygen species and prostaglandins, thereby contributing to neuronal dysfunction and cognitive decline [51]. Indoxyl sulfate has been shown to increase oxidative stress and neuroinflammatory signalling in glial cell models, supporting a direct link between retained uremic solutes and astrocyte–microglia dysfunction [41]. In the Chronic Renal Insufficiency Cohort, inflammatory markers, including high-sensitivity C-reactive protein, fibrinogen and IL-1β, were associated with a higher risk of cognitive decline, although not all inflammatory markers showed uniform associations [52]. In CKD, this inflammatory process is likely amplified by the coexistence of endothelial dysfunction, oxidative stress, alterations in the gut microbiome, and retained toxins [53,54].

### 3.5. Vascular Injury

Vascular injury provides an additional route through which uremic neurotoxicity affects cognition. CKD promotes endothelial dysfunction, vascular calcification, arterial stiffness and small-vessel disease [55,56]. These changes may impair cerebral perfusion and neurovascular coupling, which is the ability of cerebral blood flow to increase in response to neuronal activity. Studies have linked CKD with altered cerebral blood flow, reduced white matter volume and neurovascular coupling dysfunction, including hippocampal changes associated with cognitive decline [57,58,59]. Uremic toxins have been associated with worsening this process by damaging endothelial cells, reducing nitric oxide bioavailability, altering the endothelial glycocalyx and increasing blood–brain barrier permeability [14,60,61]. Brain imaging studies in CKD have linked kidney dysfunction with white matter hyperintensities, subcortical infarcts, microbleeds and impaired cerebral blood flow regulation [62,63,64,65]. These lesions preferentially affect networks involved in attention, processing speed and executive function, which are commonly impaired in CKD.

### 3.6. Glymphatic System Dysfunction

Finally, impaired brain waste clearance could possibly amplify uremic neurotoxicity. The glymphatic system is a specialised brain clearance pathway that supports the removal of proteinaceous and metabolic waste through cerebrospinal fluid–interstitial fluid exchange, particularly during sleep [66,67,68]. Limited experimental and human data indicate that sleep facilitates brain metabolite clearance, while sleep deprivation and sleep disorders impair this process [69,70,71]. Glymphatic transport can be affected in numerous states, including CKD, sleep disorders, vascular dysfunction, hypertension, inflammation and reduced physical activity. Imaging studies using diffusion tensor imaging along the perivascular space have reported glymphatic dysfunction in end-stage renal disease and early CKD, with no clear improvement after several months of dialysis in one study [72,73,74]. Reduced brain clearance could possibly lead to the accumulation of neurotoxic solutes, inflammatory mediators, and misfolded proteins, potentially promoting amyloid aggregation, neuroinflammation, and neuronal loss [75,76]. The research in this field is limited and in the early stages, but an impaired glymphatic system is an attractive research target in the field as it links CKD-related sleep disturbance, vascular disease and toxin retention to progressive cognitive decline [12,77]. As direct and robust evidence linking glymphatic dysfunction to cognitive decline in CKD patients remains limited, current findings should be interpreted as hypothesis-generating rather than definitive evidence of a causal pathway.

### 3.7. Key Signaling Pathways and Mechanism Crosstalk

Several molecular targets appear to connect the major pathological pathways involved in CKD-related cognitive vulnerability. Gut-derived indolic toxins, particularly indoxyl sulfate and indole-3-acetic acid, may act partly through aryl hydrocarbon receptor (AhR)-dependent signaling, thereby linking toxin retention with endothelial activation, oxidative stress, and inflammatory gene expression [78,79]. Oxidative stress is further amplified by activation of NADPH oxidase, depletion of glutathione, and insufficient compensatory antioxidant responses involving Nrf2-related pathways [79,80]. In parallel, inflammatory signaling pathways such as NF-κB and the NLRP3 inflammasome may promote the release of cytokines including IL-1β, TNF-α, and IL-6, supporting microglial and astrocytic activation [81]. Excitotoxic mechanisms, particularly those involving NMDA receptor activation, impaired GABAergic inhibition, calcium influx, and nitric oxide production, provide an additional link between retained guanidino compounds, homocysteine, quinolinic acid, and neuronal injury [82,83]. These pathways interact with mitochondrial dysfunction, since impaired oxidative phosphorylation, mitochondrial ROS generation, and disturbed calcium buffering can amplify both oxidative and excitotoxic injury [28]. At the vascular and barrier level, disruption of tight junction proteins such as claudin-5, occludin, and JAM-1 may increase blood–brain barrier permeability and facilitate exposure of the central nervous system to circulating toxins and inflammatory mediators [14]. Finally, reduced neurotrophic support, including altered BDNF/TrkB signaling, may further compromise synaptic plasticity and neuronal resilience [84]. Thus, CKD-related cognitive impairment is unlikely to result from a single isolated pathway, but rather from crosstalk between toxin retention, oxidative stress, mitochondrial dysfunction, endothelial injury, blood–brain barrier disruption, neuroinflammation, excitotoxicity, and impaired synaptic adaptation.

**Figure 2:**
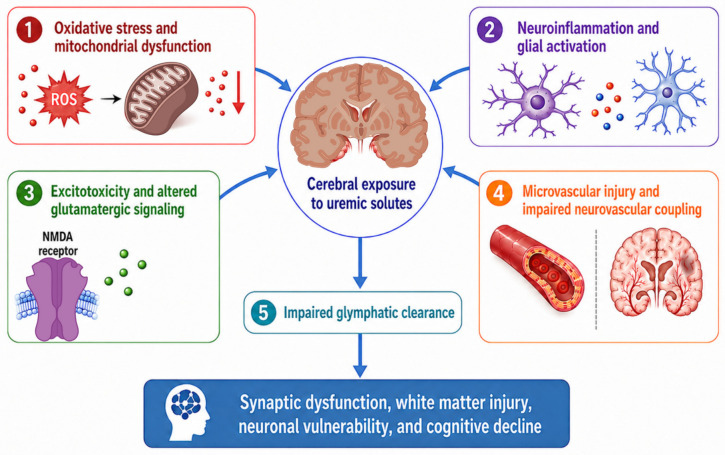
Convergent cerebral mechanisms of uremic neurotoxicity in chronic kidney disease. Cerebral exposure to retained uremic solutes converges on oxidative and mitochondrial stress, neuroinflammation and glial activation, excitotoxic glutamatergic signalling, microvascular injury, and impaired glymphatic clearance. These interacting processes promote synaptic dysfunction, white matter injury, neuronal vulnerability, and impairment of attention, processing speed, and executive function (Created in BioRender. FAJKIĆ, A. (2026) https://BioRender.com/dcfxc3).

Overall, these findings provide mechanistic support for uremic neurotoxicity, but the strength of evidence differs substantially across pathways and toxins. Experimental data are strongest for direct cellular toxicity, oxidative stress, inflammatory activation, and barrier disruption, whereas human evidence more commonly demonstrates associations between toxin burden, vascular or inflammatory markers, imaging abnormalities, and cognitive performance. There is a clear need for translational studies that combine toxin profiling, neuroimaging, inflammatory biomarkers, and domain-specific cognitive testing to identify patients at greatest risk and develop targeted interventions. These interacting pathways and their convergence on neuronal and cognitive vulnerability are summarised in Figure 2.

## 4. Clinical Phenotypes and Cognitive Profiles in Chronic Kidney Disease

Cognitive impairment is increasingly recognised as a common complication of CKD, yet it remains underdiagnosed in clinical practice [85]. As previously highlighted, the prevalence of CI among patients with CKD ranges from 10% to 40%, depending on the definition and assessment of CI and the CKD stage. Notably, CI occurs three to four times more frequently in patients with CK, particularly those with end-stage renal disease (ESRD), compared to the general population [86]. Importantly, cognitive decline often begins before kidney disease progresses to ESRD, indicating that even mild to moderate CKD may be associated with early cognitive changes [87].

Evidence suggests that both the prevalence and progression of CI are inversely related to kidney function. Large population-based studies have demonstrated that moderate CKD is associated with an increased risk of cognitive decline, with an estimated 11% higher prevalence of cognitive impairment for every 10 mL/min/1.73 m^2^ decrease in estimated glomerular filtration rate (eGFR) [85]. Furthermore, cognitive function declines more substantially when eGFR falls below 45 mL/min/1.73 m^2^, with an accelerated rate of decline observed at levels below 30 mL/min/1.73 m^2^ [88]. Although CI can occur in patients with CKD across all age groups, it is more prevalent among older adults, with older age being strongly associated with an increased risk of CI progression [89].

### 4.1. Clinical Patterns of Cognitive Impairment in CKD

Cognitive impairment in patients with CKD is associated with dysregulation across several functional domains of the brain [90]. The most pronounced deficits are observed in attention, processing speed, and, particularly, executive function [85]. Research suggests that the pattern of cognitive impairment in CKD is distinct and shares similarities with vascular dementia, which is characterised by slowed cognitive processing, prominent executive dysfunction, and relatively mild memory impairment [89]. The high prevalence of cardiovascular risk factors in CKD patients significantly contributes to the development of cognitive impairment. Additionally, ESRD may serve as a surrogate marker of accelerated atherosclerosis. Declines in frontal and executive function are commonly observed in patients with cardiovascular disease, further linking vascular pathology to cognitive deficits in CKD [91]. Executive dysfunction tends to worsen over time in patients undergoing hemodialysis and correlates with the severity of kidney impairment. Structural brain changes may underlie these deficits, as reduced frontal lobe thickness, which supports executive functions, has been reported in patients with CKD.

Patients with CKD also exhibit deficits in attention and inhibitory control, which have been associated with reduced metabolic activity in the prefrontal cortex and the locus coeruleus in the dorsal pons, as demonstrated by PET imaging. Both implicit and explicit memory appear to be affected in CKD. The storage and retrieval of explicit memories depend on the integrity of the cerebral cortex and hippocampus, as well as the activity of cholinergic neurons in the nucleus basalis of Meynert. Disruption of cholinergic–cortical interactions may therefore contribute to memory impairment. Language function is also impaired in CKD and is notable as the only cognitive domain demonstrating a linear relationship with declining eGFR. However, neuroimaging studies using MRI have not identified structural abnormalities in cortical language regions, leaving the underlying mechanisms of language dysfunction in CKD unclear [44].

The effect of CKD on visuospatial performance remains inconsistent across studies, possibly because impairments in visuospatial abilities are primarily detectable in patients with CKD stage 5. This notion is supported by neuroimaging findings, as MRI studies have not demonstrated morphological alterations in the occipital cortex, which is responsible for visuospatial processing, in patients with CKD [44].

### 4.2. Clinical Assessment of Cognitive Impairment in Patients with CKD

A comprehensive and appropriate cognitive assessment is essential for patients with suspected cognitive impairment. Even mild cognitive impairment in patients with CKD is associated with a significantly increased risk of mortality and recurrent hospitalisations. Furthermore, cognitive deficits can adversely affect decision-making capacity and self-care, potentially limiting patients’ ability to participate fully in medical decisions and adhere to prescribed treatment regimens. Given these implications, routine screening for cognitive impairment in patients with CKD is of considerable clinical importance [92].

Accurate identification of CI relies on a combination of detailed history taking, physical examination, and neuropsychological testing. Obtaining information from both the patient and family members is particularly important to establish the onset, duration, and severity of cognitive and behavioural changes, as well as the presence of functional impairments and associated symptoms [93]. However, not all global cognitive screening tools adequately assess the domains most affected in CKD, particularly executive function. Therefore, it is essential that patients with CKD undergo cognitive evaluation using tests that specifically target these commonly impaired domains. Such a targeted approach increases the likelihood of accurately capturing the full extent of cognitive impairment in this population [89].

The Mini-Mental State Examination and the Montreal Cognitive Assessment are among the most widely used cognitive screening instruments for the assessment of cognitive impairment. Although both tools evaluate global cognitive functioning, they differ considerably in their sensitivity and the cognitive domains assessed [94]. One of the principal criticisms of the MMSE is its limited evaluation of executive functioning and the relatively low complexity of its language tasks. Consequently, the MMSE may fail to detect isolated impairments in these domains, particularly during the early stages of cognitive decline. To address these limitations, the MoCA was developed as a more comprehensive cognitive screening instrument capable of identifying subtle cognitive deficits. In addition to assessing memory and orientation, the MoCA incorporates more extensive measures of executive function, language, attention, visuospatial abilities, and short-term memory.

A key distinction between the two instruments lies in their ability to detect mild cognitive impairment (MCI). The MoCA was specifically designed to identify early cognitive changes and has demonstrated substantially greater sensitivity than the MMSE. Studies have reported that the MoCA detects approximately 90% of MCI cases, whereas the MMSE identifies only 18–45%, highlighting the superior diagnostic utility of the MoCA in the early stages of cognitive decline [95]. As a result, the MoCA is generally considered the preferred screening instrument when early dementia or MCI is suspected. Furthermore, the MoCA includes more demanding tasks that require higher-order cognitive processing, particularly in the domain of executive functioning. In contrast, the MMSE places greater emphasis on orientation and memory through comparatively simple tasks, limiting its effectiveness in detecting executive dysfunction. This distinction is particularly relevant in conditions characterized by early executive deficits, such as vascular cognitive impairment.

From a clinical perspective, the MoCA is particularly appropriate for patients presenting with subjective cognitive complaints despite appearing cognitively intact during routine conversation, younger individuals with cognitive concerns, and patients in whom executive dysfunction is suspected. In contrast, the MMSE remains useful for monitoring disease progression in individuals with established moderate-to-severe dementia and in clinical settings where a brief, widely recognized cognitive screening instrument is required. It may also be preferable for patients with very low educational attainment or significant language barriers. While both instruments remain valuable tools for cognitive screening, the MoCA offers superior sensitivity for detecting mild cognitive impairment and early dementia due to its broader assessment of cognitive domains and greater emphasis on executive functioning. The MMSE continues to have clinical utility for rapid screening and longitudinal monitoring of more advanced cognitive impairment but is less effective in identifying subtle cognitive deficits [91]. Despite this, there are currently no well-validated neurocognitive tests routinely implemented in the CKD population, likely reflecting the complex and heterogeneous nature of CI in these patients [92]. It is therefore important to select cognitive assessment tools that account for common comorbidities and sensory deficits, particularly in older adults, to avoid underdiagnosis and to more accurately capture the extent of cognitive impairment [89].

Magnetic resonance imaging (MRI) provides a noninvasive method for investigating brain structural changes associated with CKD. Studies in adults with CKD have demonstrated cerebral atrophy and reduced white and gray matter density. Impaired kidney function has also been linked to glomerular small-vessel disease. In a study by Steinbach and Harshman, MRI was used to explore the hemodynamic similarities between the kidneys and cerebral vascular beds. The findings showed that reduced kidney function was associated with smaller total brain volume, decreased deep white matter volume, and a greater burden of white matter lesions. White matter hyperintensities are more commonly observed in individuals with CKD and are associated with an increased risk of cerebrovascular disease and dementia. Although macrostructural MRI markers such as white matter hyperintensities correlate with declining kidney function, microstructural alterations in white matter may represent a more sensitive indicator of cerebral injury [96].

Several mechanisms may contribute to white matter degeneration in CKD. The kidneys and brain share similar microvascular and perfusion characteristics, making both organs vulnerable to cardiovascular and hemodynamic disturbances. Additionally, hypertension, a common comorbidity in CKD, may further compromise white matter integrity. Impaired renal function is also associated with elevated circulating inflammatory mediators, which can reduce nitric oxide availability within cerebral vessels. This reduction may lead to cerebral hypoperfusion, thereby contributing to white matter damage and structural brain abnormalities [96].

Given the rising incidence of CKD, recognising and understanding cognitive dysfunction in this population has become a research priority. Characterising the extent of CI is crucial for patient care, as it can affect their capacity to make informed decisions about complex treatment regimens [97]. Since neurological complications often become clinically apparent only in advanced stages of CKD, early detection and management may help mitigate their impact over the course of the disease [93].

## 5. Biomarkers and Experimental Evidence of the Kidney–Brain Axis

The kidney–brain axis links renal impairment with cognitive decline through complex systemic and neurobiological interactions, requiring biomarkers to identify early molecular changes and interpret brain injury. Their increasing use marks a shift from descriptive clinical assessment towards a mechanism-based understanding of kidney-associated neurocognitive dysfunction [98,99,100]. Table 1 summarises key biomarkers and uremic toxins, followed by a detailed discussion of their functional and mechanistic roles.

### 5.1. Neuroaxonal Injury Markers

Neuroaxonal injury within the kidney–brain axis is most sensitively reflected by circulating neurofilament light chain (NfL), a neuron-specific cytoskeletal protein released following axonal damage [101]. Under physiological conditions, NfL is confined to axons, whereas neuronal injury leads to its release into cerebrospinal fluid and subsequently into the bloodstream [101,102]. Circulating NfL levels are influenced not only by neurodegenerative processes but also by systemic factors, particularly renal function, with studies consistently showing an inverse relationship between estimated glomerular filtration rate and plasma NfL concentrations [103,104]. Neuron-specific enolase (NSE), a glycolytic enzyme localised in neuronal cytoplasm, represents an additional marker of neuroaxonal injury. It is released into circulation following neuronal membrane disruption, particularly in hypoxia, ischemia, or acute brain injury [105,106]. Compared to NfL, NSE shows lower specificity and is more closely associated with acute neuronal damage than with chronic neurodegeneration [107,108].

### 5.2. Glial Activation and Neuroinflammation

Glial activation within the kidney–brain axis is reflected by circulating glial fibrillary acidic protein (GFAP), a marker of astrocytic activation detectable in peripheral blood. Large clinical studies show that reduced kidney function is associated with higher circulating GFAP concentrations. Population-based analyses report an inverse relationship between estimated glomerular filtration rate (eGFR) and GFAP levels, often alongside other biomarkers of neurodegeneration [109]. This relationship is supported by a systematic review and meta-analysis across diverse cohorts [103] and by studies in chronic kidney disease populations showing negative correlations between plasma GFAP and measured GFR [110]. Together, these findings indicate that circulating GFAP levels are strongly influenced by kidney function and may reflect interactions between renal dysfunction and central nervous system processes.

### 5.3. Neurotrophic Factors

Brain-derived neurotrophic factor (BDNF) is a neurotrophin involved in synaptic plasticity, neuronal survival, and memory-related processes [111]. Within the kidney–brain axis, circulating BDNF has been explored as a marker linking renal dysfunction with cognitive impairment and reduced neurotrophic support [112]. Clinical and translational studies show that BDNF levels are reduced in chronic kidney disease, suggesting impaired neurotrophic support that may increase central nervous system vulnerability. This reduction has been linked to chronic inflammation, oxidative stress, and the accumulation of uremic toxins, which are proposed to interfere with BDNF synthesis and signalling [112]. Human studies further support an association between circulating BDNF and renal function. In a cross-sectional analysis of adults without diabetes, lower fasting serum BDNF concentrations were observed in individuals with CKD, and eGFR positively correlated with fasting BDNF levels. Higher fasting BDNF was associated with a lower prevalence of CKD. In addition, several BDNF gene variants have been linked to CKD, further supporting a connection between BDNF-related signalling and renal dysfunction [113].

### 5.4. Endothelial and Blood–Brain Barrier Markers

Endothelial dysfunction and blood–brain barrier (BBB) disruption are key components of kidney–brain axis pathology in CKD. Systemic inflammation, oxidative stress, and the accumulation of uremic toxins contribute to endothelial activation and structural BBB injury, partly by degrading tight junction proteins and disrupting the integrity of the neurovascular unit [114]. Consistent with this, alterations in key tight junction proteins, including claudin-5, occludin, and junctional adhesion molecule-1 (JAM-1), have been reported in CKD, together with changes in circulating barrier-associated biomarkers, supporting BBB impairment under uremic conditions [14]. These alterations have functional consequences in humans. Patients with end-stage kidney disease show increased BBB permeability compared with healthy controls, accompanied by impaired cognitive performance. In vivo imaging studies demonstrate an inverse relationship between BBB permeability and cognitive test scores, supporting BBB dysfunction as a clinically relevant mechanism of neurological impairment in advanced CKD [115]. Further evidence comes from longitudinal studies in kidney transplant recipients, in which transplantation is associated with changes in circulating biomarkers related to the BBB and endothelial extracellular vesicle profiles. In particular, reduced levels of inflammatory particles released from the blood vessel lining (endothelial extracellular vesicles) have been observed after transplantation, suggesting a partial recovery of endothelial function and BBB integrity [14].

Several biomarkers have been proposed to characterize kidney–brain axis dysfunction, but their interpretation requires caution. Biomarkers such as NfL and GFAP are strongly influenced by renal function, raising the possibility that elevated circulating concentrations may reflect reduced renal clearance in addition to ongoing neurological injury. Furthermore, markers of blood–brain barrier dysfunction may reflect generalized endothelial injury rather than brain-specific pathology. Among currently available biomarkers, NfL is generally considered one of the most sensitive indicators of neuroaxonal injury, whereas GFAP provides complementary information regarding astrocytic activation and neuroinflammation. In contrast, NSE generally exhibits lower sensitivity and specificity, while BDNF primarily reflects impaired neurotrophic support rather than direct neuronal injury. Consequently, no single biomarker currently provides sufficient sensitivity and specificity for kidney-associated neurological injury, highlighting the need for multimarker approaches and careful adjustment for kidney function.

### 5.5. Future Biomarkers

Emerging molecular biomarkers may further improve the characterization of kidney–brain axis dysfunction. Among these, microRNAs have attracted increasing attention due to their role in regulating gene expression and vascular homeostasis. Experimental studies have demonstrated altered microRNA expression profiles in the cerebral microvasculature of CKD models [116], suggesting that CKD-induced molecular changes may affect blood–brain barrier integrity and neurovascular function. Although their clinical utility has not yet been established, microRNAs represent promising candidates for future investigation. Beyond their potential role as biomarkers, microRNAs may also contribute directly to the regulation of endothelial function, neuroinflammation, and blood–brain barrier integrity [117].

### 5.6. Uremic Toxins

Alongside proposed biomarkers, uremic toxins represent key mediators of kidney–brain axis dysfunction, linking impaired renal excretion with systemic and central nervous system effects. Although individual toxins are discussed separately for clarity, patients with CKD are exposed to multiple retained solutes simultaneously, and their neurological effects are unlikely to occur in isolation. These compounds share several pathogenic pathways, including oxidative stress, endothelial dysfunction, neuroinflammation, mitochondrial dysfunction, and blood–brain barrier impairment. The strength of evidence supporting individual toxins varies considerably, with indoxyl sulfate currently representing the most extensively studied and best-supported neurotoxic uremic solute, whereas evidence for other toxins remains more limited, indirect, or predominantly mechanistic.

#### 5.6.1. Protein-Bound Uremic Toxins

Indoxyl sulfate is considered a key uremic toxin contributing to cognitive decline in CKD, primarily through endothelial toxicity and disruption of blood–brain barrier integrity [15]. Its role is supported by both experimental and clinical studies. In rat models of CKD, including adenine-induced disease and 5/6 nephrectomy, elevated serum levels were associated with impaired cognitive performance, particularly in the novel object recognition test. Toxin overload further worsened these deficits, whereas AhR-deficient mice were protected, implicating the indoxyl sulfate-AhR pathway in brain injury associated with renal dysfunction [118]. In murine models combining nephrectomy with toxin exposure, indoxyl sulfate accumulated in blood, cerebrospinal fluid, and the prefrontal cortex, accompanied by anxiety-like and depressive-like behaviour, cognitive impairment, and markers of neuroinflammation and neuronal injury [16]. Clinical data support these findings, as higher serum levels in patients with end-stage renal disease are associated with cognitive impairment independently of amyloid-β, and with white matter microstructural changes. In vitro studies further show that indoxyl sulfate induces neuronal apoptosis through oxidative stress and caspase-dependent pathways, particularly in differentiating neuronal cells [17].

p-Cresyl sulfate, a gut microbiota-derived uremic toxin originating from aromatic amino acid metabolism, contributes to CKD progression through systemic inflammation and oxidative stress, with emerging evidence linking it to neuroinflammatory processes [18]. Mechanistically, p-cresyl sulfate has been implicated in the induction of oxidative stress through activation of NADPH oxidase, depletion of intracellular glutathione, and mitochondrial dysfunction, ultimately promoting endothelial injury and neuroinflammatory responses. Emerging evidence further suggests its involvement in the dysregulation of neurotrophic signalling pathways, including BDNF, as well as serotonergic and dopaminergic neurotransmission, thereby linking systemic uremic toxicity with central nervous system dysfunction [19]. Clinical evidence further supports the systemic vascular impact of *p*-cresyl sulfate, as elevated plasma levels independently predict ischemic stroke in patients undergoing hemodialysis, highlighting its relevance as a prognostic biomarker of cerebrovascular risk [119]. However, human studies have yielded inconsistent findings regarding its direct association with cognitive impairment, with some evidence suggesting no significant relationship between circulating *p*-cresyl sulfate levels and cognitive performance in patients with CKD, indicating potential differences in neurotoxicity among uremic solutes [20]. These inconsistencies may reflect methodological differences between studies, including relatively small sample sizes, heterogeneous CKD populations, variability in cognitive assessment methods, and important confounding factors such as age, diabetes, cardiovascular disease, and prior cerebrovascular events. Furthermore, most studies rely on single time-point measurements, which may not adequately reflect cumulative neurotoxic exposure. Differences in dialysis modality, dialysis status, residual kidney function, and the degree of uremic toxin accumulation may further contribute to variability among studies.

In addition to *p*-cresyl sulfate and indoxyl sulfate, other gut-derived indole metabolites have been increasingly implicated in the kidney–brain axis. Among them, IAA, a protein-bound uremic toxin originating from tryptophan metabolism, has emerged as a potential contributor to neurocognitive dysfunction in CKD. Clinical evidence supports a link between circulating IAA levels and impaired cognitive performance. In a multicenter cohort of hemodialysis patients, higher serum IAA concentrations were independently associated with lower scores on standardised neuropsychological tests, including the Mini-Mental State Examination (and Cognitive Abilities Screening Instrument (CASI)) [120]. These findings suggest that, like indoxyl sulfate, IAA may be a clinically relevant biomarker of cognitive decline in uremic conditions. Mechanistically, IAA has been associated with endothelial dysfunction, oxidative stress, and systemic inflammation, key processes underlying cerebrovascular damage and neurodegeneration [121]. Furthermore, its production is closely linked to gut microbiota composition, reinforcing the role of the gut–kidney–brain axis in CKD-related cognitive impairment. Although current data remain predominantly observational, the convergence of clinical and mechanistic evidence supports a contributory role of IAA in the pathophysiology of brain dysfunction in CKD.

#### 5.6.2. Metabolic (Small-Solute/Endogenous) Toxins

In addition to protein-bound uremic toxins such as p-cresyl sulfate and indoxyl sulfate, trimethylamine N-oxide (TMAO) has emerged as a key gut microbiota-derived metabolite implicated in CKD progression and systemic complications. TMAO is generated from dietary precursors, including choline and carnitine, via microbial metabolism and subsequent hepatic oxidation, and is primarily excreted by the kidneys. In CKD, impaired renal clearance leads to TMAO accumulation, which has been associated with inflammation, oxidative stress, endothelial dysfunction and fibrosis. Mechanistically, TMAO activates pro-inflammatory signalling pathways, including the NLRP3 inflammasome and NF-κB, disrupts autophagy, and induces mitochondrial dysfunction, thereby contributing to the progression of renal injury. Beyond renal effects, TMAO has been linked to vascular damage and blood–brain barrier disruption, suggesting a potential role in cognitive decline in advanced CKD stages. These findings support a broader role of TMAO within the gut–kidney–brain axis, although direct evidence in neurocognitive outcomes remains limited [122].

Beyond gut-derived metabolites such as TMAO, simple endogenous compounds also play an important role in the uremic environment. Urea, traditionally considered a low-toxicity small molecule, is increasingly recognised as a biologically active mediator of systemic and neurological complications in CKD. Experimental evidence indicates that elevated urea can accumulate in the brain and induce anxiety-like behaviour in CKD mouse models, with effects linked to oligodendrocyte precursor cell proliferation, impaired myelination in the amygdala, and altered Egr1-ERK/mTOR signalling [123]. Evidence from human post-mortem studies further demonstrates that brain urea levels are markedly elevated across multiple regions in neurodegenerative diseases, reaching concentrations comparable to those observed in uremic encephalopathy [124]. Complementary mechanistic studies extend these observations by showing that urea cycle dysregulation can directly contribute to dopaminergic neurodegeneration, partly through suppression of tyrosine hydroxylase and ODC1-dependent metabolic stress [125]. Together, these findings suggest that urea may contribute to neurotoxicity rather than acting solely as a passive retention marker.

Alongside TMAO and urea, other uremic compounds have also been implicated in neurovascular dysfunction, although the available evidence remains more limited. Lanthionine is a uremic retention product that accumulates in chronic kidney disease, particularly during hemodialysis, alongside reduced circulating hydrogen sulfide and disrupted sulfur amino acid metabolism. In vitro studies show that lanthionine inhibits hydrogen sulfide production, suggesting a mechanism contributing to vascular and metabolic toxicity in uremia [126]. Guanidino compounds, including guanidine, guanidinosuccinic acid, methylguanidine, and creatinine, are elevated in the serum, cerebrospinal fluid, and brain of patients with renal failure and have been implicated in uremic encephalopathy. Their neurotoxic effects are linked to central nervous system hyperexcitability, involving NMDA receptor activation and inhibition of GABAergic signalling pathways [30].

**Table 1:** Circulating biomarkers and uremic toxins associated with the kidney–brain axis in chronic kidney disease.

Category	Biomarker/ Toxin	Biological Source	Mechanistic Role in the Kidney–Brain Axis	Evidence Types	Key Findings	Clinical Relevance/ Limitations	Ref
Neuroaxonal injury markers	Neurofilament light chain (NfL)	Axons of large-calibre neurons	Reflects axonal injury; released following neuronal damage	Human (clinical; predominantly acute settings)	Elevated in ESRD; correlates with cognitive impairment, though influenced by renal clearance	Sensitive marker of neuroaxonal injury; interpretation confounded by renal function	[101,103,104]
	Neuron-specific enolase (NSE)	Neuronal cytoplasm	Reflects acute neuronal injury	Human (observational; acute injury studies)	Elevated in acute neuronal injury; limited utility in chronic neurodegeneration	Low specificity and limited utility in chronic conditions	[105,106]
Glial activation markers	Glial fibrillary acidic protein (GFAP)	Astrocytes	Astrocyte activation; reflects neuroinflammation	Human (clinical; population-based and CKD cohorts)	Elevated in reduced kidney function; inversely associated with eGFR; increased levels observed in CKD populations, indicating glial activation	Promising biomarker of neuroinflammation; interpretation influenced by renal function and potential reduced clearance	[103,109,110]
Neurotrophic/synaptic markers	Brain-derived neurotrophic factor (BDNF)	Circulating (serum); brain and peripheral tissues (including endothelium)	Reduced neurotrophic support; impaired synaptic function	Human (clinical; CKD cohorts) + experimental/mechanistic	Reduced circulating levels in CKD; associated with impaired neuroplasticity and endothelial dysfunction	Potential biomarker of kidney–brain axis dysfunction; influenced by metabolic and inflammatory status; not yet established as a standalone marker	[111,112,113]
Endothelial and BBB dysfunction markers	Claudin-5, Occludin, JAM-1	Endothelial tight junction complexes	Altered expression associated with BBB dysfunction	Human (clinical)	Altered tight junction protein expression associated with BBB dysfunction in CKD	Reflect structural BBB damage; limited availability in routine clinical practice	[14]
Uremic toxins /metabolic factors	Protein-bound uremic toxins						
	Indoxyl sulfate	Tryptophan metabolism (gut-derived uremic toxin)	Endothelial toxicity; BBB disruption; Neuronal apoptosis via oxidative stress	Human (clinical, ESRD patients); animal model (adenine-induced CKD mice); in vitro (SH-SY5Y differentiating neuronal cells)	Increased levels in ESRD patients with cognitive impairment; associated with white matter alterations and memory deficits	Potential biomarker linking uremic toxicity with neurodegeneration in CKD	[16,17,118]
	p-Cresyl sulfate	Gut microbiota-derived (tyrosine metabolism → p-cresol → sulfation in liver)	Induces oxidative stress via NADPH oxidase activation, glutathione depletion, and mitochondrial dysfunction; promotes endothelial inflammation (CREB/ATF1 signalling) and alters neurotrophic and neurotransmitter pathways (BDNF, serotonin, dopamine)	In vitro (endothelial, neuronal), in vivo (CKD animal models), limited human	Triggers ROS production, endothelial dysfunction, and neuroinflammation, and may contribute to alterations in neurotrophic and neurotransmitter pathways	Associated with cardiovascular and cerebrovascular risk; potential biomarker and therapeutic target in CKD	[19,119]
	Indole-3-acetic acid (IAA)	Tryptophan metabolism (gut-derived)	AhR activation; endothelial dysfunction; Oxidative stress; Inflammation	Human (hemodialysis cohort)	Higher circulating IAA levels are associated with impaired cognitive performance (MMSE, CASI) in hemodialysis patients	Potential biomarker of cognitive impairment in CKD; mechanistic link to vascular and neuroinflammatory pathways (Primarily observational evidence; lack of causal and longitudinal data)	[120,121]
	Metabolic (small-solute/endogenous) toxins						
	Trimethylamine N-oxide (TMAO)	Gut microbiota-derived metabolite (choline/carnitine metabolism)	Inflammation (NLRP3, NF-κB), oxidative stress, mitochondrial dysfunction, impaired autophagy, and endothelial dysfunction	Human CKD/ESRD cohorts + experimental animal models + mechanistic studies	Accumulates in CKD; promotes renal fibrosis and inflammation; associated with vascular dysfunction and BBB impairment	Promising biomarker and therapeutic target; direct causal role in cognitive decline not fully established	[122]
	Urea	Protein metabolism (hepatic urea cycle)	Induces osmotic stress, protein carbamylation, mitochondrial dysfunction and dysregulation of ERK/mTOR signalling; contributes to neurotoxicity and impaired myelination	Human (post-mortem brain studies); animal models (CKD, PD); in vitro	Accumulates in CKD and brain tissue; induces behavioural changes and neurodegeneration; ~3–4× increase in human brain; associated with anxiety-like behaviour and neuronal dysfunction	Levels comparable to uremic encephalopathy; increasingly recognised as an active neurotoxic mediator; causal role still under investigation	[123,124,125]
	Guanidino compounds	Amino acid metabolism	NMDA receptor activation; GABA inhibition; neurotoxicity	Human (uremia); experimental	Elevated in serum, cerebrospinal fluid, and brain in renal failure; associated with uremic encephalopathy	Contribute to neurotoxicity in CKD; limited specificity as biomarkers	[14]
	Lanthionine	Sulfur metabolite	Inhibits H_2_S production; disrupts sulfur metabolism	Human (uremia/hemodialysis); in vitro	Elevated in CKD; associated with reduced H_2_S levels and metabolic imbalance	Limited clinical evidence; primarily mechanistic	[126]
	Amino acid–derived neuroactive metabolites						
	Quinolinic acid	Tryptophan metabolism (kynurenine pathway)	Promotes neuroinflammation and excitotoxicity	Animal models (AKI, CKD; murine); human (clinical, observational); metabolomics-based studies	Elevated in CKD; associated with neuroinflammation and neuronal injury	Promising mechanistic biomarker linking renal dysfunction to brain injury; limited specificity; primarily supported by experimental and metabolomics data	[28]
	Homocysteine	Methionine metabolism (thiol-containing amino acid)	Promotes endothelial dysfunction, oxidative stress, and NMDA-mediated excitotoxicity	Human (clinical, observational); in vitro; animal models	Elevated in CKD; associated with stroke, cerebral small vessel disease, white matter lesions, and cognitive impairment	Potential modifiable risk factor (folate-dependent); lacks specificity as a biomarker; influenced by nutritional and metabolic status	[23,24]

#### 5.6.3. Amino Acid-Derived Neuroactive Metabolites

Disruption of amino acid metabolism in chronic kidney disease leads to the accumulation of neuroactive metabolites that affect neuronal and vascular function. Among these, quinolinic acid and homocysteine have emerged as key mediators linking renal dysfunction to brain injury.

Quinolinic acid is a kynurenine pathway metabolite implicated in kidney–brain axis alterations. In a recent translational study combining targeted and spatial metabolomics across murine models of acute kidney injury and chronic kidney disease, tryptophan metabolism emerged as the most altered pathway, with increased levels of kynurenine metabolites, including quinolinic acid, in plasma, kidney, and brain tissue. Elevated cortical quinolinic acid was associated with increased inflammatory markers and evidence of neuronal cell death, indicating a link between kidney dysfunction and neuroinflammation. Spatial metabolomics further localised quinolinic acid to specific brain regions, including areas adjacent to ependymal cells. Similar metabolic patterns were observed across models, and clinical data from patients with advanced CKD showed elevated plasma quinolinic acid levels, which correlated with declining kidney function and fatigue-related quality-of-life measures [28].

Homocysteine is a thiol-containing amino acid derived from methionine metabolism that accumulates in chronic kidney disease, where hyperhomocysteinemia is common. It contributes to the kidney–brain axis through both vascular and neurotoxic mechanisms. At the vascular level, homocysteine promotes a prothrombotic state and endothelial dysfunction by reducing nitric oxide bioavailability and altering fibrin structure, increasing resistance to fibrinolysis. These effects are associated with both large- and small-vessel disease, including cerebral small vessel pathology and white matter damage. Experimental and clinical data indicate that homocysteine can injure endothelial cells via oxidative stress, thereby compromising cerebrovascular integrity. Homocysteine also exerts neurotoxic effects, in part by activating NMDA receptors, contributing to excitotoxic neuronal injury. In addition, it may enhance neuroinflammation by stimulating chemokine production in monocytes and promoting leukocyte recruitment at the blood–brain barrier [23]. Experimental evidence supports this, as homocysteine administration in a murine renal ischemia–reperfusion model aggravated kidney injury and increased inflammatory cytokine expression, GFAP levels, and COX-2 upregulation in the prefrontal cortex, consistent with astrocyte activation and brain inflammation following acute kidney injury [24].

Although uremic toxins have all been implicated in kidney–brain axis dysfunction, their mechanisms and the strength of supporting evidence differ substantially. Indoxyl sulfate currently represents the most comprehensively characterized neurotoxic uremic solute, with evidence demonstrating blood–brain barrier disruption, accumulation within the central nervous system, neuroinflammation, oxidative stress, neuronal apoptosis, and cognitive impairment in both experimental models and clinical studies. In contrast, TMAO appears to exert predominantly indirect effects through systemic inflammation, endothelial dysfunction, oxidative stress, and vascular injury, whereas direct evidence of neuronal toxicity and cognitive impairment remains limited. Homocysteine occupies an intermediate position, as it contributes to both vascular injury and direct neurotoxicity through NMDA receptor-mediated excitotoxicity and neuroinflammatory mechanisms. However, unlike indoxyl sulfate, its pathogenic effects are not specific to CKD and are also observed in the general population. Collectively, current evidence suggests that indoxyl sulfate currently has the strongest experimental and clinical support as a mediator of CKD-associated neurological injury, whereas TMAO and homocysteine predominantly contribute through vascular and inflammatory pathways.

Several biomarkers have been proposed to characterize kidney–brain axis dysfunction, but their interpretation remains challenging. No single biomarker currently captures the complexity of kidney–brain axis dysfunction. Future studies will likely require multimarker approaches that integrate indicators of neuronal injury, glial activation, endothelial dysfunction, and uremic toxin burden.

## 6. Therapeutic Approaches Targeting Uremic Toxins and Their Effects

Given the limited availability of robust outcome-based evidence, current therapeutic strategies targeting uremic toxins are largely informed by mechanistic insights, pathophysiological reasoning, and extrapolation from surrogate endpoints. Within this context, a structured framework can help to conceptualise a more comprehensive approach to toxin-directed therapy in chronic kidney disease.

Therapeutic strategies targeting uremic toxins can be organised into three complementary levels: reducing toxin generation, enhancing toxin removal, and attenuating toxin-mediated biological effects [127]. This distinction is important because the therapeutic problem in chronic kidney disease is not limited to the accumulation of small water-soluble solutes but also includes protein-bound and gut-derived metabolites that are not fully controlled by conventional dialysis. Therefore, toxin-directed therapy should not be assessed solely by classical dialysis adequacy metrics, but by its ability to reduce biologically relevant toxin exposure and to modify clinically meaningful outcomes.

Conventional hemodialysis and peritoneal dialysis differ in their efficiency for removing uremic solutes, but neither fully prevents the systemic accumulation of uremic toxins. Standard dialysis is relatively effective for small water-soluble molecules, whereas protein-bound toxins such as indoxyl sulfate and p-cresyl sulfate are poorly removed because only the free fraction is available for diffusion or convection [128]. This limitation is clinically relevant because guideline-defined dialysis adequacy, usually based on urea-related metrics, does not necessarily reflect adequate control of biologically active uremic toxins. In intermittent hemodialysis, another concern is the cyclical pattern of toxin reduction and re-accumulation between sessions. Thus, even technically adequate dialysis may leave patients exposed to persistent or fluctuating toxin burdens that are not captured by routine biochemical targets [129].

Expanded hemodialysis techniques, including high-flux dialysis, online hemodiafiltration and medium cut-off membranes, have been developed to improve the removal of larger middle molecules. These modalities enhance clearance of solutes such as β2-microglobulin and selected inflammatory mediators, and they represent a rational technological improvement over conventional low-flux dialysis [130]. However, their effectiveness remains limited for protein-bound uremic toxins, which are poorly removed by diffusion and convection due to strong albumin binding. Their therapeutic relevance should therefore be interpreted cautiously: they may improve solute handling, but they should not be equated with comprehensive toxin control or proven protection from toxin-related clinical consequences. The central translational question is whether these incremental improvements in clearance translate into measurable patient benefit.

Adsorption-based approaches, including hemoperfusion with activated carbon, adsorbent cartridges, and mixed-sorbent extracorporeal systems, aim to directly bind and remove uremic toxins that are poorly cleared by conventional dialysis mechanisms. These strategies are particularly relevant for protein-bound solutes and larger inflammatory mediators. In principle, they offer a complementary mechanism to diffusion- and convection-based therapies by targeting a broader spectrum of uremic retention solutes [131,132]. However, their clinical use remains limited by cost, logistical complexity, biocompatibility concerns, and insufficient high-quality long-term outcome data demonstrating sustained benefits on hard endpoints such as mortality, cardiovascular outcomes, or cognitive function. At present, these strategies remain promising but insufficiently validated for routine use as outcome-modifying interventions.

A second therapeutic level involves reducing the intestinal production or absorption of uremic toxin precursors. Microbiome-targeted interventions, including probiotics, prebiotics, synbiotics, and dietary fibre supplementation, aim to reduce intestinal production of uremic toxin precursors, particularly those derived from protein fermentation, such as indoxyl sulfate and p-cresyl sulfate, as well as precursors of trimethylamine N-oxide. By acting upstream, gut-directed therapies could theoretically complement dialysis, which acts downstream after toxin absorption has already occurred [127,133]. However, clinical trial results remain inconsistent, likely due to heterogeneity in formulations, dosing, treatment duration, patient populations, baseline microbiome composition, dietary background and residual kidney function. Therefore, microbiome-targeted interventions should currently be viewed as biologically plausible adjuncts rather than established toxin-lowering therapies.

**Figure 3:**
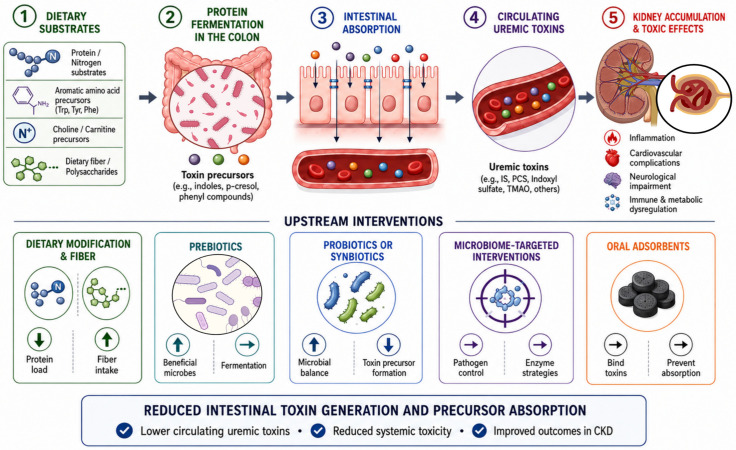
Upstream strategies to reduce intestinal generation and absorption of uremic toxins in chronic kidney disease. Dietary precursor modulation, fibre-based interventions, prebiotics, probiotics or synbiotics, microbiome-targeted approaches, and oral adsorbents may reduce intestinal generation or absorption of uremic toxin precursors. These approaches target the gut–kidney interface and should currently be considered adjunctive strategies pending outcome-based clinical validation (Created in BioRender. FAJKIĆ, A. (2026) https://BioRender.com/kviv26h).

Oral adsorbents represent another upstream strategy. AST-120, a charcoal-based spherical adsorbent, has been widely studied for its ability to bind intestinal precursors of uremic toxins and reduce their systemic absorption. Its rationale is strong, especially for gut-derived solutes such as indoxyl sulfate [134]. Nevertheless, clinical findings have been inconsistent, and its role remains uncertain outside selected settings [135,136]. The experience with AST-120 illustrates a broader challenge in the field: reducing toxin exposure may be mechanistically sound, but clinical benefit depends on patient selection, timing of intervention, residual renal function, adherence and outcome selection. The principal upstream strategies aimed at reducing intestinal generation and absorption of uremic toxin precursors are summarised in Figure 3.

Pharmacological strategies in this area focus on mitigating the downstream effects of uremic toxins rather than their removal. These interventions do not primarily reduce toxin burden, but aim to limit the biological consequences of toxin exposure. Anti-inflammatory, antioxidant and endothelial-protective approaches may theoretically reduce toxin-related systemic injury. Agents acting through oxidative stress pathways, including Nrf2-related mechanisms, have been discussed in this context, although their clinical applicability remains uncertain. Importantly, such therapies should be considered adjunctive rather than toxin-specific. They may reduce vulnerability to uremic injury, but they do not solve the upstream problem of toxin generation or the extracorporeal problem of insufficient clearance.

Several experimental or early-stage strategies also deserve attention. Albumin-binding displacement has been proposed as a method to increase the free fraction of protein-bound toxins, thereby enhancing dialytic removal. Preservation or enhancement of residual kidney function may also reduce toxin accumulation, particularly in earlier stages of CKD or among dialysis patients with remaining diuresis [137]. However, these approaches remain largely investigational, and their safety, feasibility, and long-term clinical relevance have not yet been established.

The clinical application of toxin-directed therapies should be adapted to the stage of CKD, reflecting the evolving pathophysiology of uremic toxin accumulation. In early CKD (stages G1–G3), preservation of kidney function remains the most effective strategy to limit toxin accumulation, while dietary interventions, gut microbiota modulation, and optimization of kidney-protective therapies may reduce the generation of gut-derived uremic toxins [138]; however, progressive loss of renal clearance necessitates combining upstream interventions with strategies aimed at improving extracorporeal toxin removal, although current dialysis techniques remain insufficiently effective for highly protein-bound solutes such as indoxyl sulfate and *p*-cresyl sulfate [139]. In patients receiving maintenance dialysis, technological advances including expanded hemodialysis, online hemodiafiltration and adsorption-based therapies should therefore be regarded as complementary approaches rather than definitive solutions for toxin control.

Importantly, current evidence demonstrates a persistent discrepancy between biochemical efficacy and clinical benefit. Although several interventions reduce circulating concentrations of indoxyl sulfate and *p*-cresyl sulfate, randomized and observational studies have not consistently demonstrated parallel improvements in cognition or other patient-centred neurological outcomes. This inconsistency likely reflects the multifactorial pathogenesis of cognitive impairment in CKD, where uremic toxins represent only one component among vascular disease, chronic inflammation, oxidative stress, anemia and metabolic disturbances. Furthermore, most available clinical studies are limited by relatively small sample sizes, heterogeneous study populations, short follow-up duration and the use of surrogate biochemical markers rather than validated neuropsychological assessments [140]. To date, no adequately powered randomized clinical trial has demonstrated that selective reduction in indoxyl sulfate or p-cresyl sulfate alone results in a significant improvement in cognitive performance in patients with CKD. Consequently, toxin-lowering interventions should currently be considered biologically plausible but not yet evidence-based neuroprotective therapies. Future randomized controlled trials integrating biochemical, imaging and standardized cognitive endpoints are required before stage-specific recommendations targeting cognitive preservation can be made.

Overall, therapeutic targeting of uremic toxins remains mechanistically compelling but clinically incomplete. At present, the field is largely driven by mechanistic rationale, pathophysiological extrapolation, and surrogate biochemical endpoints rather than robust outcome-based evidence. The major limitation is therefore not the lack of plausible interventions, but the absence of consistent demonstration that toxin-directed strategies translate into meaningful clinical benefit. Future research should move beyond sole reliance on solute concentrations and instead incorporate validated cognitive, functional, and patient-centred outcomes as primary endpoints. Well-designed studies will be essential to establish a coherent, evidence-based framework for toxin-targeted therapy in chronic kidney disease. Until then, current and emerging interventions should be interpreted as part of a developing conceptual framework rather than established neuroprotective strategies.

## 7. Future Directions and Clinical Implications

Cognitive decline is a prevalent but still underappreciated complication of CKD, with substantial implications for morbidity, treatment complexity, and patient-centred outcomes. The core problem is that its presentation in CKD is insidious and fluctuating; deficits are frequently masked by fatigue, depression, sleep disturbance, anaemia, or uremic symptoms. Structured cognitive assessment is rarely integrated into standard care pathways, and there is a clear need to promote routine screening. Instruments such as the Montreal Cognitive Assessment (MoCA) are practically advantageous given their greater sensitivity to executive dysfunction compared with brief memory-oriented tools [141]. Cognitive screening should not serve as a gatekeeping tool for advanced therapies, but rather as a means of identifying patients who may require adapted education, caregiver engagement, simplified therapeutic regimens, and structured multidisciplinary support.

Cognitive screening in CKD should also be adapted to the level of medical care. In tertiary hospitals and academic nephrology centres, especially those managing advanced CKD, dialysis, transplantation, and complex comorbidity, cognitive assessment should be structured and multidisciplinary. Patients with CKD stage 4–5, dialysis dependence, transplant evaluation, recurrent hospitalisation, suspected poor adherence, functional decline, or caregiver-reported cognitive changes should undergo screening with instruments sensitive to executive dysfunction, such as the MoCA, supplemented when appropriate by tests of attention, processing speed, and executive function. Abnormal results should prompt further evaluation for reversible contributors, including anaemia, sleep disturbance, depression, medication burden, metabolic derangements, and vascular risk factors, and referral for neuropsychological, neurological, geriatric, or psychiatric assessment when indicated.

In regional nephrology clinics and dialysis units, a pragmatic approach may be more feasible. Cognitive screening could be performed at baseline in high-risk patients and repeated periodically, for example, annually or when there is a change in clinical status, adherence, functional independence, or dialysis planning. In these settings, brief instruments such as MoCA, Mini-Cog, or locally validated cognitive tools may be used according to available time and staff training. The main goal is not to establish a formal dementia diagnosis, but to identify patients who need simplified education, caregiver involvement, medication review, adherence support, or referral for specialist assessment.

In primary care and grassroots medical settings, screening should be based on case-finding rather than universal detailed assessment. Priority should be given to older patients with CKD, patients with advanced CKD or albuminuria, those with vascular comorbidities, repeated missed appointments, difficulty managing medication, loss of independence, or family concern about cognition. Very brief tools, functional questions, and caregiver input may help identify patients requiring referral. Such a tiered model allows cognitive screening to be clinically useful without overburdening lower-resource settings, while ensuring that patients with suspected impairment are directed toward appropriate specialist evaluation and support.

In non-dialysis CKD, therapeutic strategies targeting uremic toxins should focus on gut-derived, protein-bound solutes. These include indoxyl sulfate, p-cresyl sulfate, indole-3-acetic acid, trimethylamine-N-oxide, and advanced glycation end-products, all of which are poorly cleared by native kidney function and conventional dialysis alike. There is growing evidence that these toxins contribute to cognitive impairment through several overlapping mechanisms: oxidative stress, endothelial dysfunction, blood–brain barrier disruption, microglial activation, mitochondrial injury, and chronic neuroinflammation [142]. Future treatment approaches will likely require a multimodal strategy that combines dietary and microbiome modulation, intestinal adsorption, preservation of residual kidney function, and vascular protection. Personalised toxin profiling, together with cognitive phenotyping, endothelial biomarkers, neuroimaging, and microbiome analysis, could pave the way for precision nephrology.

Furthermore, several conclusions can be drawn with different levels of certainty. First, the most firmly supported conclusion is that cognitive impairment is common in CKD and has clear clinical relevance, particularly because it affects executive function, attention, processing speed, treatment adherence, decision-making capacity, and patient-centred outcomes. Second, uremic toxin retention provides a biologically plausible and clinically meaningful framework linking impaired renal clearance with endothelial dysfunction, oxidative stress, neuroinflammation, mitochondrial injury, blood–brain barrier disruption, and neuronal vulnerability. However, these mechanisms should be interpreted as contributory and biologically plausible rather than definitively causal in humans. Third, several emerging concepts, including toxin-guided cognitive risk stratification, biomarker-based prediction, glymphatic dysfunction, microbiome-directed neuroprotection, and precision toxin profiling, remain promising but insufficiently validated. These areas should therefore be viewed as hypothesis-generating research directions rather than established clinical practice. A clearer distinction between established evidence, plausible mechanisms, and unverified concepts is essential for advancing the kidney–brain axis field without overstating the current level of evidence.

Research now needs to move beyond association toward a mechanistic and translational understanding. Particularly, future longitudinal studies and interventional trials are needed to determine whether reducing toxin burden or modifying inflammatory, endothelial, mitochondrial, or blood–brain barrier pathways can alter cognitive trajectories in CKD. Interventional strategies should be developed in parallel, including optimisation of cardiovascular risk profiles, reduction in uremic toxin burden, and targeted cognitive rehabilitation [86]. Robust clinical trials with cognitive endpoints remain scarce, which is a major unmet need and an area where real progress can be made.

High-resolution mass spectrometry has expanded the range of uremic solutes that can be identified, reshaping our understanding of the “uremic toxin landscape” and yielding new insights into CKD-associated systemic and neurovascular toxicity [143]. Clinical translation remains hampered by challenges in standardisation, but ongoing toxin profiling should eventually support the integration of these findings into risk prediction and therapeutic decision-making.

## Data Availability

No new data were created or analyzed in this study.

## Abbreviations

The following abbreviations are used in this manuscript:

CKD	Chronic Kidney Disease
CI	Cognitive impairment
eGFR	Estimated glomerular filtration rate
BBB	Blood–Brain barrier
TMAO	Trimethylamine-N-oxide
NMDA	N-methyl-D-aspartate
ESRD	End-stage renal disease
MMSE	Mini-Mental State Examination
MoCA	Montreal Cognitive Assessment
NfL	Neurofilament light chain
NSE	Neuron-specific enolase
GFAP	Glial fibrillary acidic protein
BDNF	Brain-derived neurotrophic factor
JAM-1	Junctional adhesion molecule-1
IAA	Indole-3-acetic acid
CASI	Cognitive Abilities Screening Instrument
MCI	Mild cognitive impairment
MRI	Magnetic resonance imaging
